# Data visualization, bar naked: A free tool for creating interactive graphics

**DOI:** 10.1074/jbc.RA117.000147

**Published:** 2017-10-03

**Authors:** Tracey L. Weissgerber, Marko Savic, Stacey J. Winham, Dejana Stanisavljevic, Vesna D. Garovic, Natasa M. Milic

**Affiliations:** From the ‡ Division of Nephrology and Hypertension and; the ¶ Division of Biomedical Statistics and Informatics, Mayo Clinic, Rochester, Minnesota 55905 and; the § Department of Medical Statistics and Informatics, Medical Faculty, University of Belgrade, Belgrade 11000, Serbia

**Keywords:** bar graphs, data visualization, interactive graphics, open science, transparency

## Abstract

Although bar graphs are designed for categorical data, they are routinely used to present continuous data in studies that have small sample sizes. This presentation is problematic, as many data distributions can lead to the same bar graph, and the actual data may suggest different conclusions from the summary statistics. To address this problem, many journals have implemented new policies that require authors to show the data distribution. This paper introduces a free, web-based tool for creating an interactive alternative to the bar graph (http://statistika.mfub.bg.ac.rs/interactive-dotplot/). This tool allows authors with no programming expertise to create customized interactive graphics, including univariate scatterplots, box plots, and violin plots, for comparing values of a continuous variable across different study groups. Individual data points may be overlaid on the graphs. Additional features facilitate visualization of subgroups or clusters of non-independent data. A second tool enables authors to create interactive graphics from data obtained with repeated independent experiments (http://statistika.mfub.bg.ac.rs/interactive-repeated-experiments-dotplot/). These tools are designed to encourage exploration and critical evaluation of the data behind the summary statistics and may be valuable for promoting transparency, reproducibility, and open science in basic biomedical research.

## Introduction

Interactive graphics may be valuable tools for promoting transparency, reproducibility, and open science (1) at a time when these factors are highly valued (2–4). Funding agencies, scientific journals, and investigators are concerned about the lack of transparency and reproducibility of published data (5, 6), especially in preclinical research (2). The lack of availability of raw data as well as suboptimal data presentation and statistical analysis practices contribute to problems with validating and reproducing study results (7–10). Several recent papers have examined the potential value of interactive graphics as tools for improving data presentation in scientific publications (1, 11, 12). We recently proposed that offering interactive alternatives to common static figures may be an effective strategy for improving data visualization in small-sample size studies (1). Our proof-of-concept tool for creating interactive line graphs illustrates the potential of interactive graphics to transform the reader from a passive consumer into an active participant by facilitating exploration of published data. The present paper builds upon this foundation by introducing a free, web-based “interactive dotplot” tool for creating customized interactive graphics. The tool is designed as an interactive alternative to the bar graph, which is routinely used to compare values of a continuous variable across different study groups.

## Why focus on small-sample size studies?

Small-sample size studies are common in basic biomedical, preclinical, and translational research. These studies influence decisions regarding which prevention and treatment strategies advance to the expensive and time-consuming clinical trials process, thereby potentially influencing future clinical practice. The National Institutes of Health recently highlighted preclinical research as being particularly susceptible to irreproducibility (2). Preclinical animal studies typically have small samples (*e.g. n* = 8/group) (13). In other basic science fields, most studies have fewer than 15 subjects/group; sample sizes of 3–6 subjects or samples per group are common (14). Promoting transparent data presentation and advancing open science for small-sample size studies should be part of a broader strategy to improve reproducibility in scientific research. Although the interactive dotplot tool is designed for small-sample size studies, most of its functions are also effective with larger samples.

## Moving beyond bar graphs: What can interactive alternatives add?

**Figure 1.**
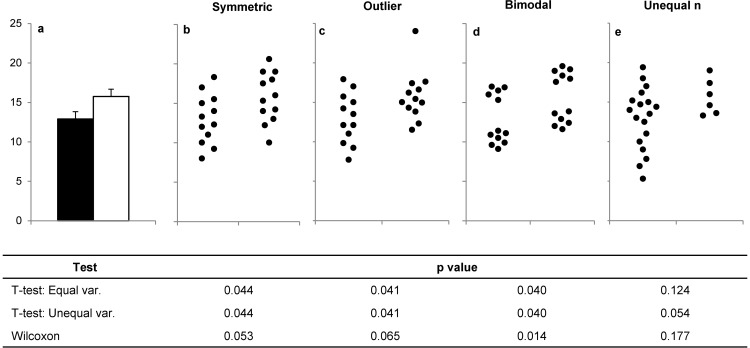
**Many different distributions can lead to the same bar graph.** The full data may suggest different conclusions from the summary statistics. The means and S.E. values for the four example datasets shown in *b–e* are all within 0.5 units of the means and S.E. values shown in the bar graph (*a*). *p* values were calculated in R statistical software (version 3.0.3) using an unpaired *t* test, an unpaired *t* test with Welch's correction for unequal variances, or a Wilcoxon rank sum test. In *b*, the distribution in both groups appears symmetric. Although the data suggest a small difference between groups, there is substantial overlap between groups. In *c*, the apparent difference between groups is driven by an outlier. *d* suggests a possible bimodal distribution. Additional data are needed to confirm that the distribution is bimodal and to determine whether this effect is explained by a covariate. In *e*, the smaller range of values for group 2 may simply be due to the fact that there are only three observations. Additional data for group 2 would be needed to determine whether the groups are actually different. *var*, variance. Adapted from Weissgerber *et al.* (14) under a creative commons license.

The reliance on non-transparent bar graphs to present data from small-sample size studies is of particular concern. Bar graphs are designed for counts and proportions, yet they are routinely used to present continuous data from small-sample size studies. Traditionally, the height of the bar shows the group mean while the error bar shows the S.E. or S.D. A recent systematic review reported that 86% of papers published in the top 25% of physiology journals used bar graphs to present continuous data (14). Graphics that show the data distribution, such as univariate scatterplots and box plots, were rarely used (14). This is problematic, as many different datasets can lead to the same bar graph, and the actual data may suggest different conclusions from the summary statistics (Fig. 1) (14–17). Many journals, including the *Journal of Biological Chemistry*, *PLOS Biology*, *eLife*, and *Nature*, have recently addressed this problem by implementing new guidelines that encourage or require authors to select figures that show the data distribution (10, 18–26).^4^ Investigators have launched initiatives encouraging other journals to implement similar policies (27),^4^ and numerous blog posts and webpages have encouraged better data presentation practices (28–30).^4^ However, bar graphs continue to be a widely accepted strategy for presenting continuous data in many fields.

**Figure 2.**
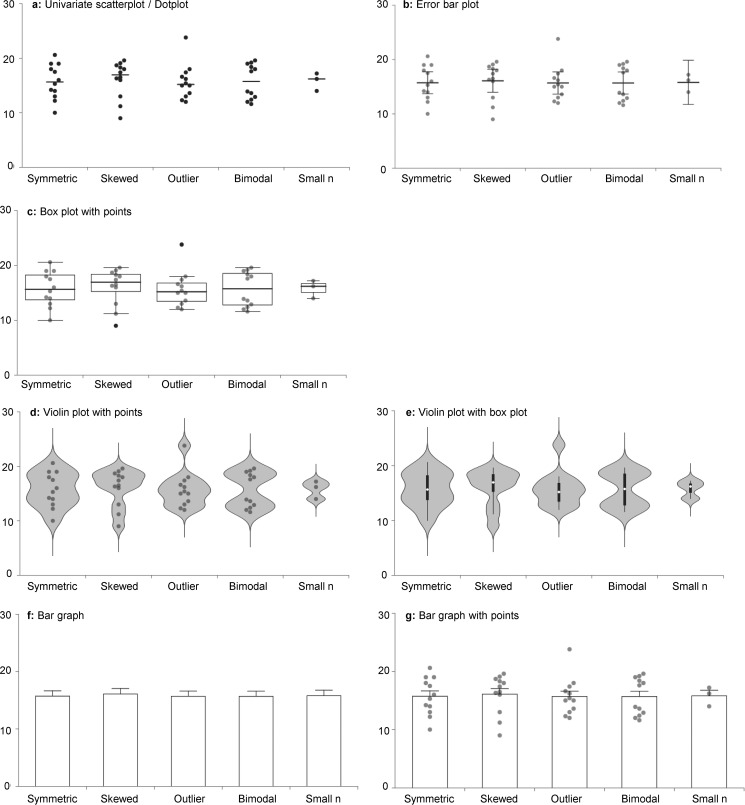
**Different figures emphasize different aspects of the data.** This figure shows some examples of graphs that can be created using the interactive dotplot tool and illustrates how different figures emphasize different aspects of the data (see Box 1). *a*, univariate scatterplot showing group means. *b*, error bar plot showing the mean and 95% confidence interval, with data points. *c*, box plot with data points. The *center line* of the *box* represents the group median, whereas the *top* and *bottom* of the *box* represent the 75th and 25th percentiles. *Whiskers* are extended to the most extreme data point that is no more than 1.5 × interquartile range from the edge of the box (Tukey style). *Black dots beyond* the *whiskers* represent outliers. *d* and *e*, violin plots estimate the data distribution by using a kernel density function. The violin plot includes an adjustable smoothing parameter, which controls how closely the “violin” shape follows the distribution shown by the data points. Data points (*d*) or a box plot (*e*) can be added to the center of the violin plot. *f* and *g*, bar graph showing mean and S.E., with (*g*) or without (*f*) data points. The interactive dotplot tool includes bar graphs for educational purposes; however, they are not recommended for use in scientific publications.

This paper introduces a free, web-based tool for creating an interactive alternative to the bar graph (http://statistika.mfub.bg.ac.rs/interactive-dotplot/).^4^ The interactive dotplot allows one to easily compare values of a continuous variable across study groups by viewing different graphs that show the data distribution, including univariate scatterplots or dotplots, box plots, and violin plots. Individual data points or summary statistics may be overlaid on the graphs. Each of these traditionally static graphics has different strengths and limitations (Fig. 2 and Box 1); hence, there are many datasets for which no single graph is optimal. Different types of graphs may be needed, depending on the characteristics of the data as well as the interests of the person viewing the graph. The interactive dotplot tool allows authors with no programming expertise to quickly create interactive graphics that are designed to increase transparency while encouraging one to explore and critically evaluate the empirical data behind the summary statistics.

Box 1. Different graphs emphasize different aspects of the data

- Effective figures for scientific publications should:
  1. Immediately convey information about the study design
  2. Illustrate important findings
  3. Allow the reader to critically evaluate the data
- Univariate scatterplot or dotplots showing the raw data points are the best option for very small samples (*n* ≤ 10 per group, Fig. 2*a*), as the summary statistics shown in other graphs are only meaningful when there are enough data to summarize. These plots can also provide valuable information for larger samples.
- Box plots summarize the data distribution by showing five characteristics. The box represents the interquartile range (IQR) and includes all values between the 25th and 75th percentile of the sample. The line inside the box presents the median. The whiskers are most often defined as the most extreme data points that are not outliers (Tukey style). Outliers are shown as individual data points outside the whiskers. Box plots allow one to quickly compare the entire data distribution across different groups and identify groups with more variability. Skewed distributions and outliers can be identified on box plots; however, bimodal distributions cannot be detected unless the data points are shown (Fig. 2*c*).
- Violin plots are very effective for showing the shape of the data distribution in medium or large samples. Skewed distributions, bimodal distributions, and samples with outliers each have distinctive shapes (Fig. 2, *d* and *e*).
- Bar graphs are routinely used to present continuous data in small-sample size studies despite the fact that they do not provide the information needed to critically evaluate the data (Fig. 2*f*). This impedes a pivotal part of the scientific process. Adding data points to the bar graph highlights another limitation; bar graphs arbitrarily assign importance to the bar height (17), instead of focusing attention on how the difference between means or medians compares with the range of values observed in the sample (Fig. 4). The *y*-axis typically starts at zero and ends just above the highest error bar. This can distort the perception of the range of observed values by including low values that never occur in the population (*Zone of Irrelevance*), while excluding values above the highest error bar that may be common in the population (*Zone of Invisibility*).

**Figure 3.**
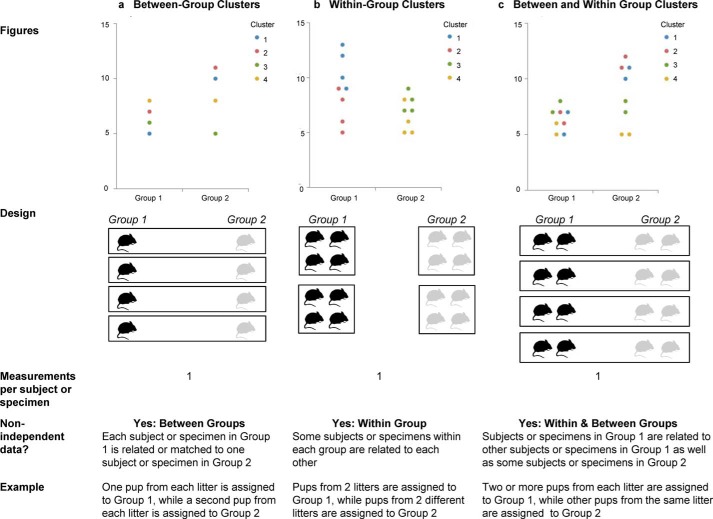
**Clustered data.** The interactive dotplot tool can create graphs for studies with clustered of non-independent data. The *colors* on the graph show whether the study includes between-group clusters, within-group clusters, or between- and within-group clusters. This allows one to determine whether any observed differences are consistent across all clusters. Adapted from Weissgerber *et al.* (21).

Additional features of the interactive dotplot facilitate visualization of subgroups or clusters of non-independent data. Even a well-designed static figure may not contain all of the information needed to explain a particular dataset. Whereas showing subgroups is useful in many situations, this feature may be particularly valuable given recent National Institutes of Health requirements to consider sex as a biological variable (31). Clusters of non-independent data, such as technical replicates or mice from the same litter, are common in basic science research, yet these data are often inappropriately analyzed (32). The different types of clustered designs shown in Fig. 3 each require different approaches to data visualization and statistical analysis. Information about the presence and type of clustering is rarely apparent in static graphics but can be easily visualized and explored using the interactive dotplot tool.

Readers can explore the tool by entering or uploading their own data or using the two example datasets posted on the homepage (subgroup example and clustered data example described in Box 2). The four key functions of the interactive dotplot tool are as follows.

Box 2: Examples

These examples are designed to illustrate the functions of the “interactive dotplot” and “interactive repeated experiments” tools and show how the tools can be used to increase transparency for different types of datasets. Interactive graphics for each of the examples below are posted on the home page for the tool. Users can explore the graphics by right-clicking to download and save the datasets, and then uploading them into the tool. More detailed instructions on how to use the tools to create interactive graphics or explore datasets are found under the instructions link for each tool.

**Subgroups example**

**Dataset:** Urinary levels of Biomarker 1 and Biomarker 2 were assessed in wild-type and knockout mice. The experiment included both male and female animals.

**Interactive dotplot:** The interactive dotplot shows that knockout mice have lower values of Biomarker 1 than wild-type mice (Fig. S1 in interactive dotplot). Color-coding the subgroups reveals that the proportion of male mice was higher in the knockout group, compared with the wild-type group. Furthermore, viewing the subgroups side-by-side reveals that the male mice have lower concentrations of Biomarker 1 than female mice (Fig. S2 in interactive dotplot). The lower concentrations of Biomarker 1 in knockout mice may be partially explained by the greater number of males in this group. In contrast, Biomarker 2 does not appear to differ between wild-type and knockout mice, and similar values are observed in males and females (Fig. S3 in interactive dotplot).

**Clustered data example**

**Dataset:** This simulated dataset includes four litters of mice. Four mice are selected from each litter. Two mice from each litter are assigned to receive a supplement in drinking water, and the remaining two mice from each litter are assigned to the control group (normal drinking water). The example data file includes values for two biomarkers that were measured in plasma after 8 weeks of exposure to normal or supplemented drinking water.

**Interactive dotplot:** When viewing the dotplot, selecting the option to color-code clusters immediately reveals that the study used a between- and within-groups clustered design with two observations from each cluster in each group. The dotplot for Biomarker 1 suggests that although higher values are observed in the Supplement group, compared with Control group, there is considerable overlap between groups (Fig. C1 in the Dotplot menu of the interactive graphic). Furthermore, the effect of the supplement is not consistent across litters. The higher values in the Supplement group compared with the Control group are observed in litters 1 and 2, but not litters 3 and 4. The graph for Biomarker 2 reveals that values in the Supplement group are lower than those in the Control group for all four litters (Fig. C2). Using the data reduction option to show the mean or median for each cluster confirms this finding (Fig. C3).

**Repeated experiments example**

**Dataset:** In each of three repeated experiments, cells are exposed to a drug or placebo for 5 days. The concentrations of Biomarkers 1 and 2 are measured in the culture media each day.

**Interactive graphic:** Use the Repeated Experiments tool (http://statistika.mfub.bg.ac.rs/interactive-repeated-experiments-dotplot/) to view this graph. Although the initial graph shows a dotplot, one can add trend lines to create a spaghetti plot by checking the box labeled “Connect time points/conditions for individual experiments.” Exploring the interactive dotplot for Biomarker 1 reveals that there is little overlap between the drug and placebo groups. Viewing the results of each experiment individually shows that concentrations in the drug group are higher than in the placebo group from day 2 to day 5. This can be seen by using the small multiples button or the checkboxes next to each experiment (Fig. R1). In contrast, Biomarker 2 does not show this pattern (Fig. R2). There are no clear differences between the drug and placebo group; values frequently overlap, and any observed differences are not consistent across experiments.

### 1. Viewing different types of graphs

**Figure 4.**
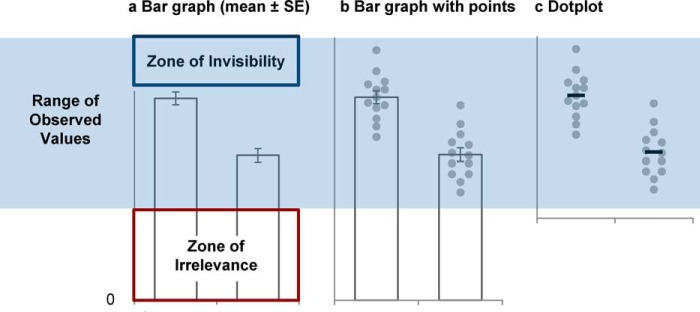
**Anatomy of a bar graph.** Bar graphs arbitrarily assign importance to the height of the bar rather than focusing attention on how the difference between means compares to the range of values observed in the sample. *a*, the *bar height* represents the mean, and the *error bars* each represent one S.E. The *y* axis starts at zero and ends *just above* the *highest error bar. b*, adding data points reveals that the *y* axis scale distorts one's perception of the range of observed values. The bar graph in *a* includes low values that never occur in the sample (*Zone of Irrelevance*) and excludes values above the highest error bar that are observed in the sample (*Zone of Invisibility*). *c*, the dotplot emphasizes how the difference between means compares with the range of values observed in the sample. The *y* axis includes all observed values.

The interactive tool allows one to view a dotplot, box plot, and violin plot. Additional options make it easy to add data points and different types of summary statistics to the graph. Although bar graphs are not recommended for presenting continuous data (Fig. 4), they are included as an educational tool. Comparing bar graphs with figures that show the data distribution highlights the limitations of bar graphs and allows one to identify situations in which they may be particularly misleading (Fig. 2).

### 2. Examining subgroups

Graphs include options to display observations and summary statistics from different subgroups of participants (*e.g.* males and females) in different colors. If a study examines C-reactive protein levels in obese *versus* lean participants, for example, one could use the interactive subgroup options to show data points and summary statistics from men and women in different colors. Subgroups can also be positioned side-by-side.

### 3. Graphing clustered or correlated data

Interactive graphics that include data points allow users to graph clusters of non-independent data, such as technical replicates or mice from the same litter. Data points from each cluster appear in a different color, making it easy to determine whether the study includes between-group, within-group, or between- and within-group clusters (Fig. 3). This information is critical when determining which statistical techniques can be used to analyze the data (33). When viewing dotplots, investigators can use the “data reduction” option to show only the mean or median of each cluster. This simple technique is frequently used to analyze clustered data in small studies.

### 4. Focusing on groups, clusters, or subgroups of interest

Groups, clusters, or subgroups can be displayed individually, making it easier to focus on interesting features in the dataset.

Additional options allow the user to customize the graph axes and labels, view the interactive graphic in a color-blind–safe color palette, and download .tiff files of static graphs for print publication. An .xml file of the interactive graphic dataset, including saved static graphs, can be included in the data supplement of the published paper. Readers can explore the interactive or saved static graphs by uploading the .xml file into the website.

This free, web-based tool is posted on a publically accessible website for the University of Belgrade Medical School (Belgrade, Serbia) (http://statistika.mfub.bg.ac.rs/interactive-dotplot/).^4^ User data are stored in a temporary file on the web server, which is deleted after the user leaves the website. The site does not store or archive any data.

A second version of the tool, designed for laboratory studies with repeated experiments, is available at http://statistika.mfub.bg.ac.rs/interactive-repeated-experiments-dotplot/.^4^ This tool, for example, might be used to examine data from three repeated experiments, which compare cell counts each day for 5 days in cells exposed to drug *versus* placebo treatment (Box 2). The tool allows investigators to determine whether results are consistent across experiments by examining data from each individual experiment. Trend lines can be added to facilitate visualization of changes over time or across conditions. Checking the “small multiples” box allows one to view a series of small graphs, each of which highlights the results of a different experiment.

## Conclusions

Developing user-friendly tools that create interactive alternatives to common static figures may be a simple and effective strategy for promoting widespread use of interactive visualizations in scientific research. The interactive dotplot tool, which includes univariate scatterplots, box plots, and violin plots, allows one to quickly create an interactive alternative to the bar graph. Additional features facilitate visualization of subgroups or clusters of non-independent data. We hope that this free, web-based tool will advance open science by making the underlying data an integral part of the scientific publication. Interactive alternatives to static graphs have the potential to improve transparency and transform scientific publications from static reports into interactive datasets. These figures allow the reader to explore the dataset rather than being constrained to a single graph presented by the study authors.

## Author contributions

T. L. W. and N. M. M. conceptualization; T. L. W., M. S., D. S., and N. M. M. software; T. L. W. and N. M. M. supervision; T. L. W. visualization; T. L. W. and N. M. M. writing—original draft; T. L. W. and N. M. M. project administration; T. L. W., M. S., S. J. W., D. S., V. D. G., and N. M. M. writing—review and editing; S. J. W. and V. D. G. resources.

## Footnotes

4 Please note that the JBC is not responsible for the long-term archiving and maintenance of this site or any other third party hosted site.

## References

[1] Weissgerber T. L., Garovic V. D., Savic M., Winham S. J., and Milic N. M. (2016) From static to interactive: transforming data visualization to improve transparency. PLoS Biol. 14, e1002484 27332507 10.1371/journal.pbio.1002484 PMC4917243

[2] Collins F. S., and Tabak L. A. (2014) Policy: NIH plans to enhance reproducibility. Nature 505, 612–613 24482835 10.1038/505612a PMC4058759

[3] Alsheikh-Ali A. A., Qureshi W., Al-Mallah M. H., and Ioannidis J. P. (2011) Public availability of published research data in high-impact journals. PLoS One 6, e24357 21915316 10.1371/journal.pone.0024357 PMC3168487

[4] Wadman M. (2013) NIH mulls rules for validating key results. Nature 500, 14–16 23903729 10.1038/500014a

[5] Vaux D. L. (2012) Research methods: know when your numbers are significant. Nature 492, 180–181 23235861 10.1038/492180a

[6] (2016) Take the long view. Nat. Med. 22, 1 26735395 10.1038/nm.4033

[7] Halsey L. G., Curran-Everett D., Vowler S. L., and Drummond G. B. (2015) The fickle *P* value generates irreproducible results. Nat. Methods 12, 179–185 25719825 10.1038/nmeth.3288

[8] Krumholz H. M. (2015) The end of journals. Circ. Cardiovasc. Qual. Outcomes 8, 533–534 26555127 10.1161/CIRCOUTCOMES.115.002415 PMC5322965

[9] Weissgerber T. L., Garovic V. D., Milin-Lazovic J. S., Winham S. J., Obradovic Z., Trzeciakowski J. P., and Milic N. M. (2016) Re-inventing biostatistics education for basic scientists. PLoS Biol. 14, e1002430 27058055 10.1371/journal.pbio.1002430 PMC4825954

[10] Teare M. D. (2016) Transparent reporting of research results in eLife. Elife 10.7554/eLife.21070 PMC5017861 27612386

[11] Hayes B. (2012) Pixels or perish. Am. Scientist 10.1511/2012.95.106

[12] Ellis D. A., and Merdian H. L. (2015) Thinking outside the box: developing dynamic data visualizations for psychology with Shiny. Front. Psychol. 6, 1782 26648881 10.3389/fpsyg.2015.01782 PMC4664644

[13] Holman C., Piper S. K., Grittner U., Diamantaras A. A., Kimmelman J., Siegerink B., and Dirnagl U. (2016) Where have all the rodents gone? The effects of attrition in experimental research on cancer and stroke. PLoS Biol. 14, e1002331 26726833 10.1371/journal.pbio.1002331 PMC4699644

[14] Weissgerber T. L., Milic N. M., Winham S. J., and Garovic V. D. (2015) Beyond bar and line graphs: time for a new data presentation paradigm. PLoS Biol. 13, e1002128 25901488 10.1371/journal.pbio.1002128 PMC4406565

[15] Saxon E. (2015) Beyond bar charts. BMC Biol. 13, 60 26246239 10.1186/s12915-015-0169-6 PMC4526301

[16] Pallmann P., and Hothorn L. A. (2016) Boxplots for grouped and clustered data in toxicology. Arch. Toxicol. 90, 1631–1638 26438403 10.1007/s00204-015-1608-4

[17] (2014) Kick the bar chart habit. Nat. Methods 11, 113 24645190 10.1038/nmeth.2837

[18] PLOS Biology (2016) Submission guidelines: data presentation in graphs. http://journals.plos.org/plosbiology/s/submission-guidelines#loc-data-presentation-in-graphs

[19] Fosang A. J., and Colbran R. J. (2015) Transparency is the key to quality. J. Biol. Chem. 290, 29692–29694 26657753 10.1074/jbc.E115.000002 PMC4705984

[20] International Journal of Primatology (2015) Instructions for authors. http://www.springer.com/life+sciences/evolutionary+%26+developmental+biology/journal/10764?detailsPage=pltci_2474567

[21] Weissgerber T. L., Garovic V. D., Winham S. J., Milic N. M., and Prager E. M. (2016) Transparent reporting for reproducible science. J. Neurosci. Res. 94, 859–864 27377972 10.1002/jnr.23785 PMC5330667

[22] Journal of Biological Chemistry (2015) Instructions for authors. http://www.jbc.org/site/misc/ifora.xhtml#experimental

[23] Journal of Neuroscience Research. (2016) Author guidelines. http://onlinelibrary.wiley.com/journal/10.1002/(ISSN)1097-4547/homepage/ForAuthors.html#anchor8

[24] Kidney International (2017) Guide for authors. http://www.kidney-international.org/content/authorinfo#idp1694800

[25] George C. H., Stanford S. C., Alexander S., Cirino G., Docherty J. R., Giembycz M. A., Hoyer D., Insel P. A., Izzo A. A., Ji Y., MacEwan D. J., Sobey C. G., Wonnacott S., and Ahluwalia A. (2017) Updating the guidelines for data transparency in the *British Journal of Pharmacology*: data sharing and the use of scatter plots instead of bar charts. Br. J. Pharmacol. 174, 2801–2804 28801996 10.1111/bph.13925 PMC5554317

[26] (2017) Towards greater reproducibility. Nature 546, 8 28569823 10.1038/546008a

[27] (2016) #BarBarPlots. https://barbarplots.github.io/index.html

[28] Koyama T. Beware of dynamite. http://biostat.mc.vanderbilt.edu/wiki/pub/Main/TatsukiRcode/Poster3.pdf

[29] Rousselet G. (2016) One simple step to improve statistical inference. in Basic Statistics: Simple Steps to Improve Statistical Analyses in Neuroscience & Psychology. https://garstats.wordpress.com/2016/03/09/one-simple-step-to-improve-statistical-inferences/

[30] Jambor H. (2016) #BarBarPlots. http://thenode.biologists.com/barbarplots/photo/

[31] National Institutes of Health. (2015) NOT-OD-15–102 Consideration of sex as a biological variable in NIH-funded research. http://grants.nih.gov/grants/guide/notice-files/NOT-OD-15–102.html

[32] Lazic S. E. (2010) The problem of pseudoreplication in neuroscientific studies: is it affecting your analysis? BMC Neurosci. 11, 5 20074371 10.1186/1471-2202-11-5 PMC2817684

[33] Galbraith S., Daniel J. A., and Vissel B. (2010) A study of clustered data and approaches to its analysis. J. Neurosci. 30, 10601–10608 20702692 10.1523/JNEUROSCI.0362-10.2010 PMC6634702

